# Growth of Silicon Nanosheets Under Diffusion-Limited Aggregation Environments

**DOI:** 10.1186/s11671-015-1138-2

**Published:** 2015-10-30

**Authors:** Jaejun Lee, Sung Wook Kim, Ilsoo Kim, Dongjea Seo, Heon-Jin Choi

**Affiliations:** Department of Materials Science and Engineering, Yonsei University, 120-749 Seoul, South Korea

**Keywords:** Silicon nanosheets, CVD, Epitaxial growth, Diffusion-limited aggregation

## Abstract

The two-dimensional (2D) growth of cubic-structured (silicon) Si nanosheets (SiNSs) was investigated. Freestanding, single-crystalline SiNSs with a thickness of 5–20 nm were grown on various Si substrates under an atmospheric chemical vapor deposition process. Systematic investigation indicated that a diffusion-limited aggregation (DLA) environment that leads to dendritic growth in <110> directions at the initial stage is essential for 2D growth. The kinetic aspects under DLA environments that ascribe to the dendritic and 2D growth were discussed. Under the more dilute conditions made by addition of Ar to the flow of H_2_, the SiNSs grew epitaxially on the substrates with periodic arrangement at a specific angle depending on the orientation of the substrate. It reveals that SiNSs always grew two dimensionally with exposing (111) surfaces. That is thermodynamically favorable.

**Electronic supplementary material:**

The online version of this article (doi:10.1186/s11671-015-1138-2) contains supplementary material, which is available to authorized users.

## Background

Two-dimensional (2D) nanomaterials, such as graphene and transition metal dichalcogenides (TMDs), have been intensively researched because of their excellent physical and chemical properties [1]. For example, graphene is characterized by an excellent Young’s modulus, high thermal conductivity, and high electron mobility. Likewise, monolayers of TMDs exhibit direct band gap transitions that result in field-effect transistor (FET) devices with high on/off ratios [2]. Other applications for graphene and TMDs have also been investigated, including flexible and transparent devices, high-speed transistors, optical devices, sensors, and energy-harvesting devices. However, these 2D nanomaterials are not compatible with current silicon (Si)-based complementary metal oxide semiconductor (CMOS) processes, which are critical for the fabrication of devices. The large-scale synthesis, large domain size, surface residue, doping, and air stability of these 2D nanomaterials should also be addressed to exploit their potential.

Regarding this, 2D Si nanomaterials should be of interest and are expected to have novel physical and chemical properties as well as CMOS compatibility. A few studies have investigated 2D silicon nanomaterials. For example, monolayers of Si, also known as silicene, exhibit new physical and chemical properties, including a Dirac cone band structure, the quantum spin Hall effect, and band gap opening by surface modification [3–5]. Recently, silicene-based FET devices have shown ambipolar charge transport properties, which are promising for electronic devices [6]. However, silicene has dangling bonds on its surface because of sp^3^ bonds. Therefore, its surface is reactive and easily destroyed in an ambient environment. Other hybrid structures that consist of silicene and organic materials have also been synthesized and characterized [7–9]. However, these structures are not freestanding 2D silicon and, thus, are difficult to use to fabricate devices.

We have previously reported the growth of freestanding silicon nanosheets (SiNSs) composed of a few layers of Si [10, 11]. It was revealed that the SiNSs were very stable in ambient atmosphere. We also demonstrated that the SiNSs exhibited a thickness-dependent optical band gap opening in the range of 1.8 to 3.2 eV. This indicates that the SiNSs have a great potential for use as CMOS compatible 2D nanomaterials in many optoelectronic devices. However, 2D growth of nanosheet with cubic-structured Si is still unclear. In this study, we grew SiNSs on (100), (110), and (111) Si substrates under controlled gas flow conditions and investigated the 2D growth mechanism of SiNSs.

## Methods

Silicon wafers were cleaned using acetone followed by 2-propanol (IPA). For epitaxial growth, the native oxide of the silicon wafers was eliminated using buffered oxide etch (BOE) for 7 min. Diced silicon wafers were located at the center of the furnace. To purge the furnace, H_2_ and Ar gases were used. Next, the temperature of the furnace was elevated to 1000 °C for 30 min and liquid SiCl_4_ was bubbled by H_2_ gas at 1–50 sccm. H_2_ and Ar carrier gases were flowed at 500–3000 sccm and 0–3000 sccm, respectively. The hot zone temperature of the furnace was maintained at 1000 °C for 10–180 min and then cooled to room temperature.

## Results and Discussion

**Fig. 1:**
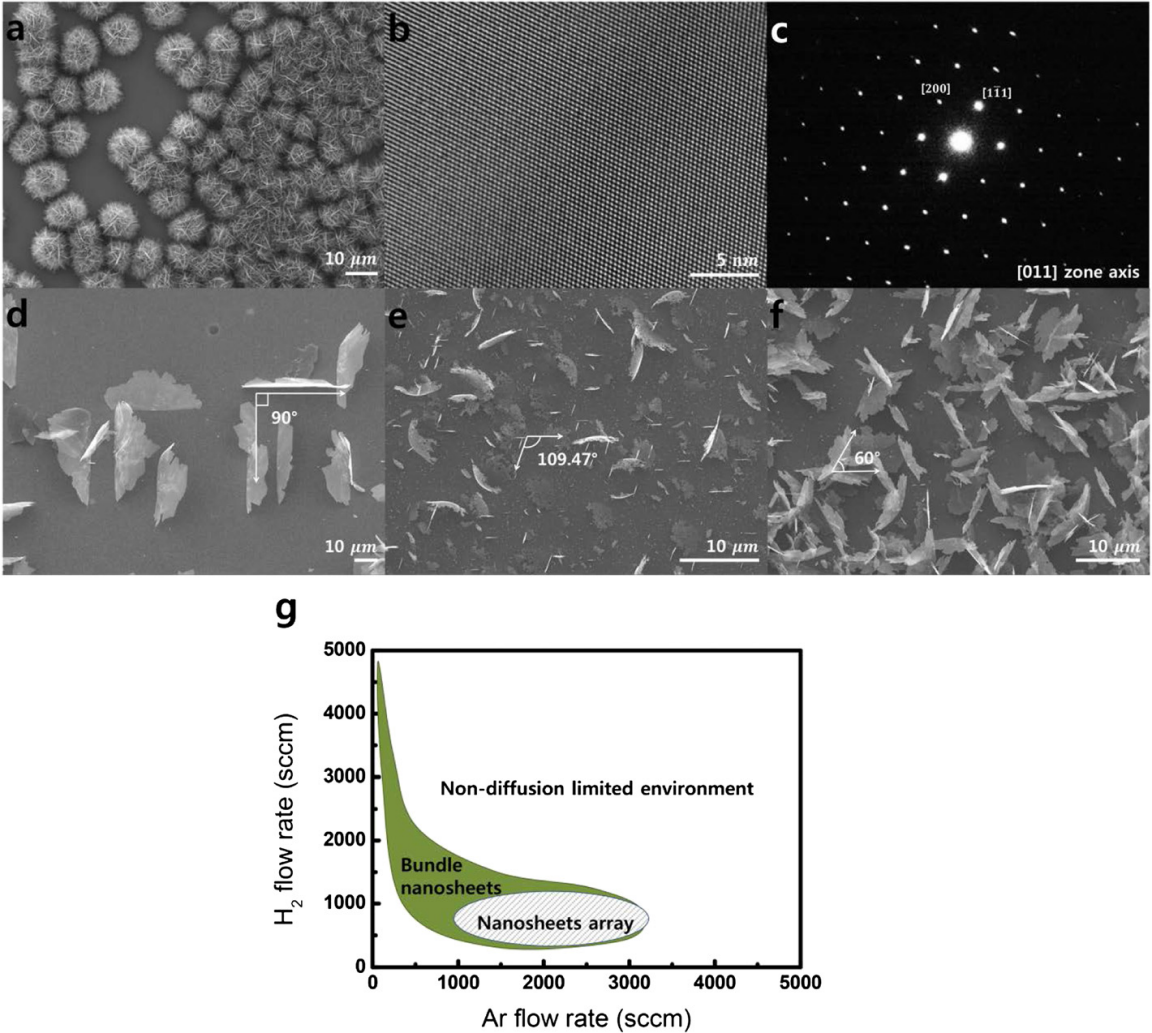
SiNSs grown by controlling the flow rate of H_2_. **a** SEM image of spherically congregated SiNSs. **b** HRTEM image. (**c**) SAED pattern of SiNSs showing single crystallinity. **d–f** Top view SEM images of controlled epitaxial SiNSs on (100), (110), and (111) substrates, respectively. **g** Conditions for the formation of arrays and bundles of SiNSs

The SiNSs were grown under a high flow rate of H_2_ gas, which led to the 2D nucleation and growth of the cubic Si crystals (Fig. 1a). The SiNSs has a (111) surface orientation and thickness of nanometer scale, as reported previously [10, 11]. The SiNSs were single crystals, which were observed using high-resolution transmission electron microscopy (HRTEM) (Fig. 1b). The selected area electron diffraction (SAED) pattern showed that the SiNSs were perfect single crystals (Fig. 1c). Atomic force microscopy showed that the SiNSs had a few (111) layers with a thickness of 5–20 nm (Additional file 1: Figure S1).

We grew SiNSs under various flow rates of H_2_ and found that SiNSs grew under dilute concentration of SiCl_4_. In our atmospheric chemical vapor deposition (CVD) system, SiCl_4_ was bubbled with H_2_ at 2–20 sccm and the H_2_ carrier gas was flowed at 1000–3000 sccm. In this environment, the ratio of the SiCl_4_ to carrier gas (Ar and H_2_) of the reaction was 5.3 × 10^−6^, which was calculated by measuring the amount of SiCl_4_ liquid consumed. This was 1000 times lower than that of other groups which grow silicon thin films (Table 1) [12–17].

**Table 1:** Mole fraction of SiCl_4_/ H_2_ + Ar. Our mole fraction of SiCl_4_ is 1000 times lower than that of other groups who grew Si thin film using SiCl_4_

Number	Mole fraction of SiCl_4_/H_2_ + Ar	Reference
1	0.000005348	Our data
2	0.005–0.02	[16]
3	0.3	[17]
4	0.02–0.31	[18]
5	0.005–0.035	[19]
6	0.02	[20]
7	0.167	[21]

The growth of SiNSs was then investigated on the various (100), (110), and (111) Si substrates (Fig. 1d–f). Under H_2_ flow, the SiNSs grew in a bundle shape and showed no dependency on the type of substrates. By introducing the high flow rate of Ar, however, we found that freestanding SiNSs were epitaxially grown on (100), (110), and (111) silicon substrates (Fig. 1d–f). This indicates that the growth of SiNSs can be controlled by introducing Ar gas into the H_2_ flow. To grow SiNSs epitaxially, the H_2_ flow rate should be between 500 and 1000 sccm and the Ar flow rate should be over 1000 sccm when SiCl_4_ is blown by H_2_ at 2 sccm. If the flow rate of SiCl_4_ is higher, the Ar and H_2_ flow rates should be increased. It is noted that the growth mechanism consisted of dendritic growth and filling process is working under any gas flow conditions [10, 11].

In Fig. 1g, the growth condition for SiNSs is summarized. Shaded box indicate condition for growing SiNSs array shown in Fig. 1d–f, and green area shows condition of growth of bundle shape of SiNSs presented in Fig. 1a. H_2_ and Ar gas with high flow rates both dilute the concentration of the Si source. Meanwhile, H_2_ is involved in the decomposition reaction with SiCl_4_, which provides Si to the growth sites as follows: SiCl_4_ + 2H_2_ = Si + 4HCl. However, Ar is not involved in the decomposition of SiCl_4_ and, thus, dilutes the Si source more effectively without additional reactions. This makes it possible for 2D growth without any mingling with each other and enables SiNSs to grow individually and epitaxially. In summary, by diluting the reactant condition with Ar gas, the epitaxial growth of SiNSs could be achieved on the substrates.

Our systematic investigation of the gas flow conditions confirms that the very dilute precursor concentration is essential for the 2D growth of SiNSs. Such a crystal growth condition is classified as a diffusion-limited aggregation (DLA) environment, wherein the rate of crystal growth is dominated by the diffusion rate of the reactants [18]. The main feature of DLA environments is strong anisotropic, dendritic growth due to the low concentration of precursors [19]. Accordingly, cubic-structured Si in this study grew dendritically in [110] direction at the early stages and leads to two-dimensional growth of dendritic networks that is essential for the growth of SiNSs.

**Fig. 2:**
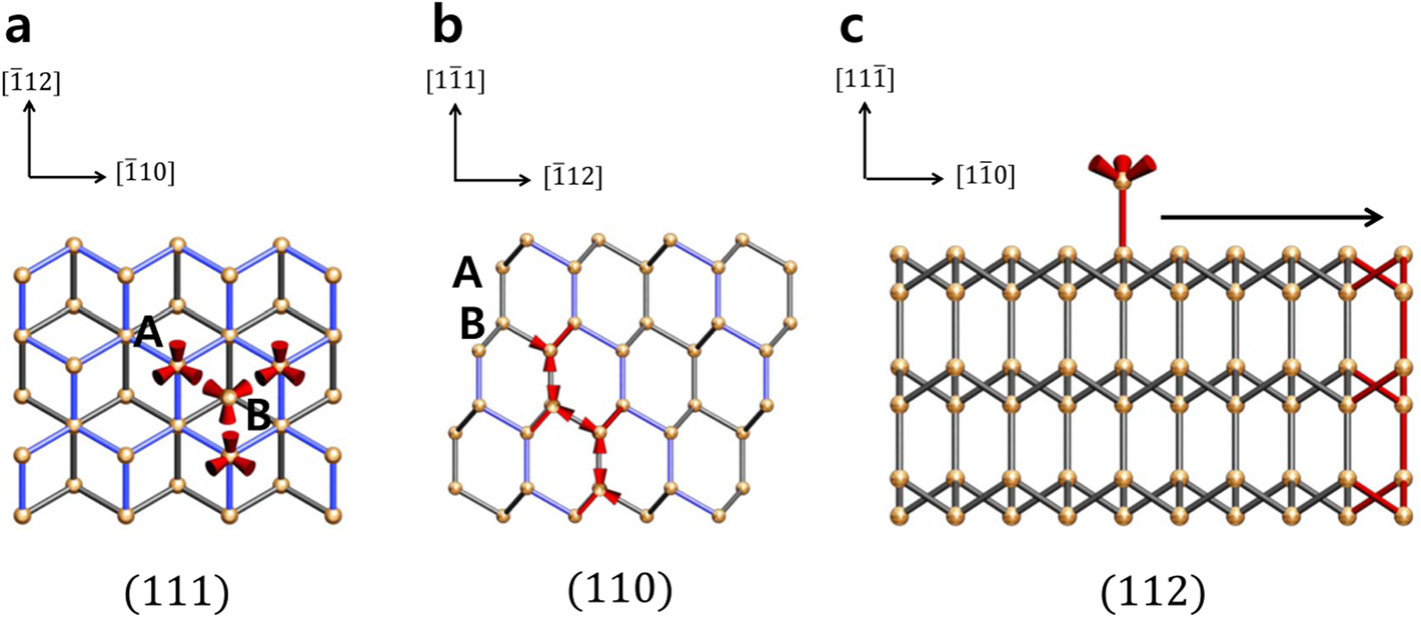
Schematic images of the surface of the SiNSs showing the adsorption process. *Gray bonds* represent the back bonds of the SiNSs, and *blue bonds* represent the bonding of the surface atoms. **a** The adsorption process on the (111) surface. The atoms on site A have bonds with the surface atoms. The atoms on site B bond with atoms on site A. In a diffusion-limited environment, it is difficult for atoms on site B to bond with atoms on site A and could not form the (111) layer because of the diffusion of atoms on site A. **b** On the (110) surface, atoms on site A act as a step site. Therefore, atoms adsorbed on site B have two bonds. **c** Projection view towards the [112] direction shows the diffusion of adsorbed atoms on the (111) surface to the (110) surface

The other crystal orientation effect in DLA environment could also ascribe to the dendritic growth. In this environment, the diffusion of the reactant to the substrate is very slow and, as a result, there are few atoms adsorbed on the surface of the silicon nucleus. Especially, on the (111) silicon surface, adsorbed atoms on site A (Fig. 2a) that have single bonds with the surface atoms are unstable in high temperature such as our growth temperature of 1000 °C, which means that surface mobility of adatoms on (111) surface is high enough to diffuse to other sites (Fig. 2c). These onefold coordinated adsorbed atoms diffuse to higher energy sites such as in the <100> and <110> directions, which have threefold coordinated sites [20]. Adsorbed atoms on site A would be stabilized when they bond with adsorbed atoms on site B. However, it is difficult for an absorbed atom to bond with two adjacent atoms on site B because of the fast diffusion of absorbed atoms on the (111) surface in high temperature. Moreover, the Si (111) 7 × 7 reconstruction has been reported in Si thin film growth system [21–24]. The number of dangling bonds on 7 × 7 reconstructed surface is only 19, which could enhance diffusion of adatoms [25]. This results in the suppression of growth towards the [111] direction. These behaviors also appeared at island growth of thin film by molecular beam epitaxy (MBE) system [25]. High surface mobility of adsorbed atom makes a flat island, and diffusion to high coordinated sites makes the growth of an island. Also, the stability of edge formation towards [112] has been reported in Si thin film growth [22–24]. On the other hand, on the (110) surface that is perpendicular to the (111) surface, adsorbed atoms on site B have bonds with the surface atoms and form bonds with other adsorbed atoms on site A (Fig. 2b). These two bonds stabilize the adsorbed atom and fix it so it does not diffuse. Therefore, its growth proceeds continuously. Ultimately, as forming nucleus on the (111) surface is inhibited, dendrite growth towards (110) is only proceeded two dimensionally, which induces nanostructure to form a sheet shape. As a result, suppressed growth on (111) and dendritic growth on (110) which is the perpendicular directions of [111] result in the exposure of the (111) surface of the SiNSs. It is noted that the exposing of the (111) surface is also thermodynamically favorable by exposing the surface with the lowest surface energy [26].

This 2D growth mechanism can be further confirmed by close investigation of the epitaxial growth behavior of the SiNSs on the various substrates. On the (100) substrate, the SiNSs were arranged perpendicular to each other (Fig. 1d). Their attach line on the substrate had [110], [−110], [−1–10], and [1–10] directions, which are perpendicular to the [100] direction. These four directions result in a rectangular pattern. On the (110) substrates, there were two SiNSs that had different tilt angles with the substrate. Perpendicularly grown SiNSs formed a parallelogram pattern and made an angle of 109.47°. Their attach lines had [1–12] and [−112] directions, which are perpendicular to the [110] direction. The other SiNSs had a tilt angle of 35.26°, which formed on the [1–10] attach line and made a line pattern (Fig. 1e). On the (111) substrates, the SiNSs arranged in a triangular pattern, which had an angle of 60° and formed on [101], [110], and [011] attach lines (Fig. 1f).

**Fig. 3:**
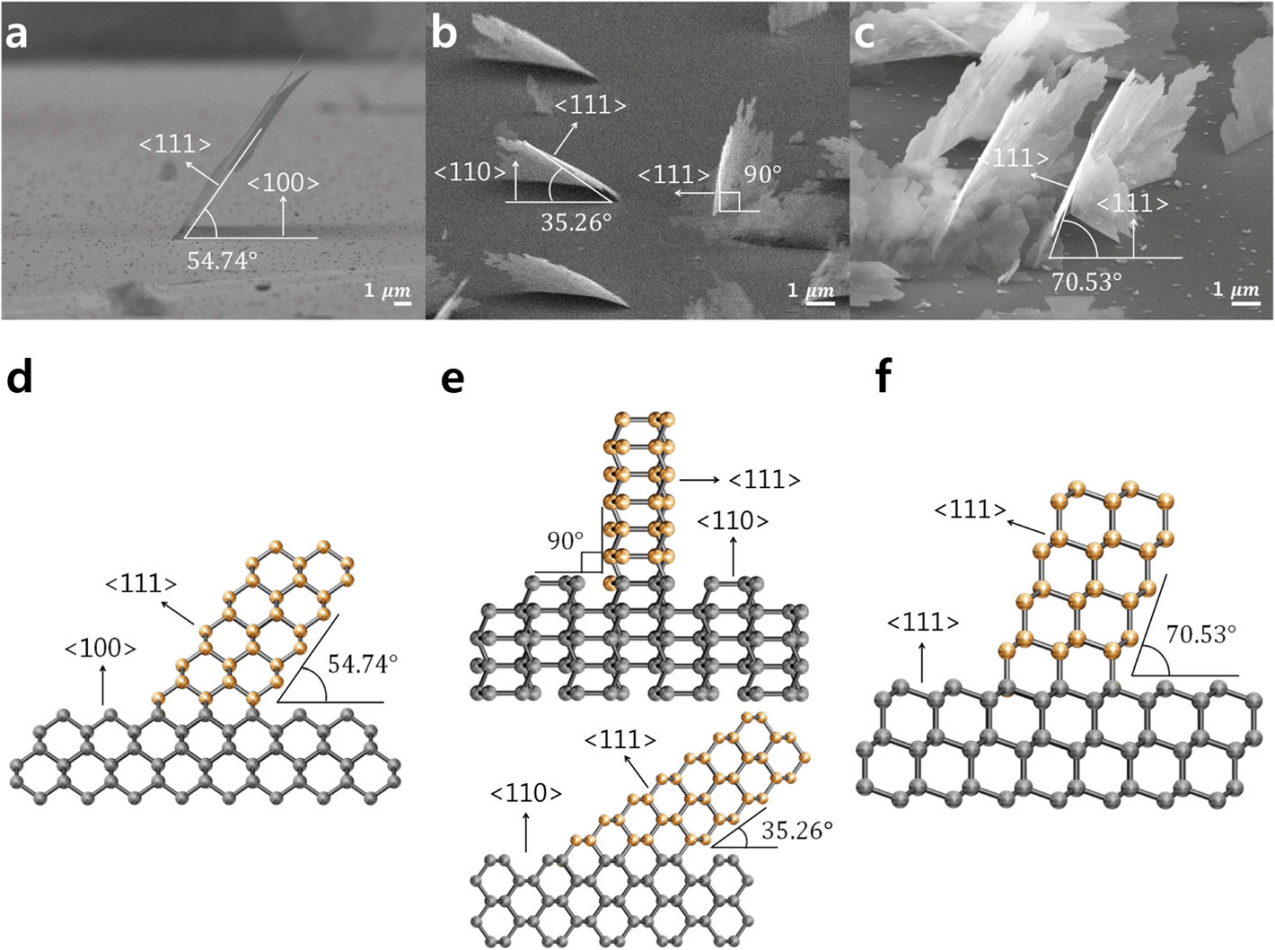
Cross-sectional view of SiNSs. **a–c** SEM images of epitaxial SiNSs presenting the angle with the substrates depending on the orientation of the substrates. **d–f** Schematic images of the side projection view of the epitaxial SiNSs. This shows continuous atomic stacking has angles and an exposed surface that match those of the SiNSs in **a–c**

**Fig. 4:**
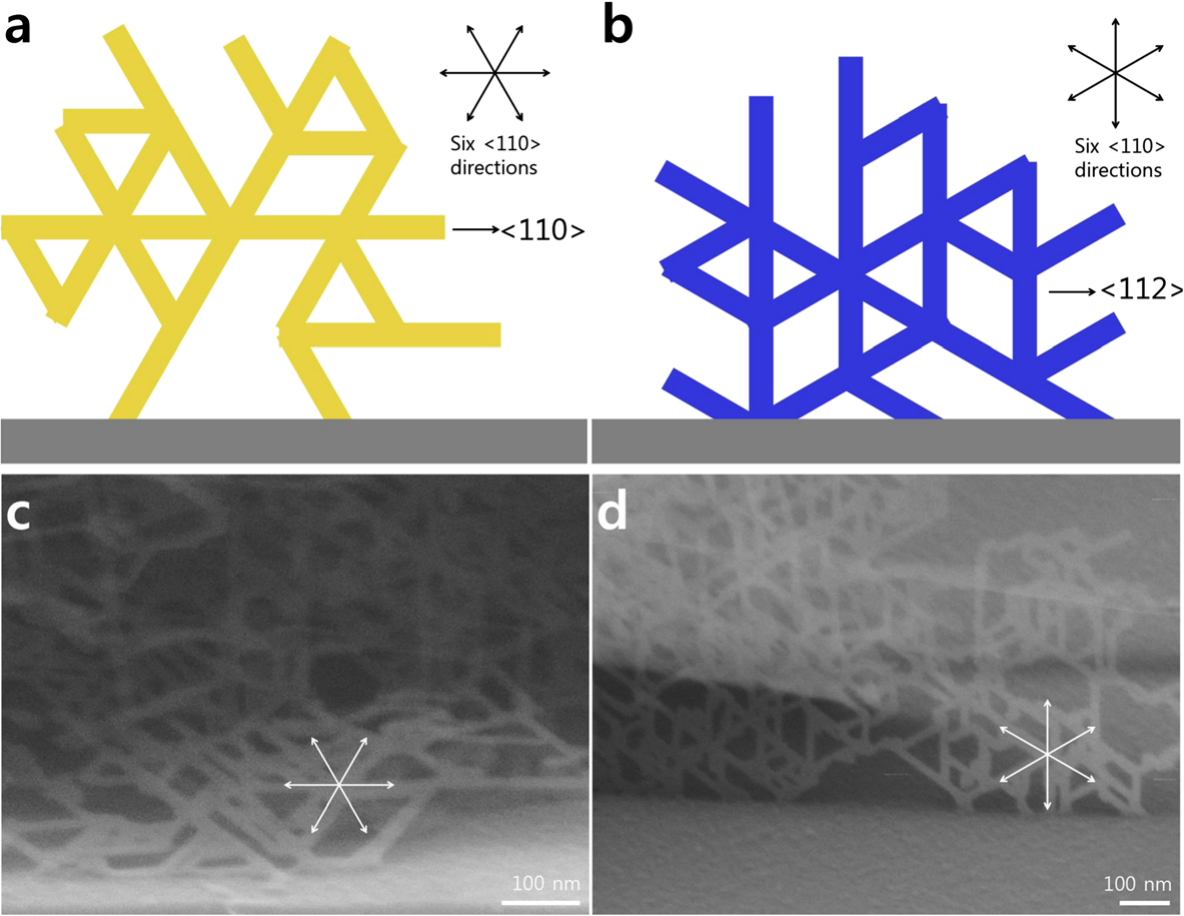
Growth behaviors of non-vertically and vertically grown SiNSs. **a**, **c** Schematic and SEM images showing the dendritic growth of tilted SiNSs, respectively. Each dendrite grows towards the six directions of <110> and has <110> side projections. **b**, **d** Schematic and SEM images of the dendritic growth of vertical SiNSs, respectively. These SiNSs have <112> side projections

In the cross-sectional view, the SiNSs had an angle of 54.74° with the (100) substrate (Fig. 3a). In Fig. 3d, the side projection schematic image of the SiNSs on the (100) substrate shows continuous stacking of atoms, which exposes the {111} surface and the {110} side projections. It clearly indicates that the orientation of the SiNSs and the angle with the (100) substrates matches with the scanning electron microscopy (SEM) image shown in Fig. 3a. On the (110) substrate, there were two different types of growth. One has an angle of 35.26° with a substrate that has {110} side projections. The other is perpendicular to the substrate and has {112} side projections (Fig. 3b, e). On the (111) substrate, the SiNSs had an angle of 70.53° and had {110} side projections (Fig. 3c, f). Meanwhile, it should be noted that the tilted SiNSs had {110} side projections while the vertically grown SiNSs had {112} side projections. This difference is seen during the branching process that occurs during dendritic growth of the SiNSs (Fig. 4). Moreover, the tilted SiNSs have horizontal branches (Fig. 4a, c). However, vertically grown SiNSs have vertical branches (Fig. 4b, d). This occurs because the growth direction of the branches is fixed in the six <110> directions and the surface of the SiNSs is also fixed in the {111} direction. The specific angles that are found between the SiNSs and the tilt angles that are observed according to the orientation of the substrates clearly show the characteristics of epitaxial growth. It should be noted that the growth direction of the SiNSs is determined by the (111) surface and that the growth angle to the substrate is altered according to the substrate orientation to expose the (111) surface. This indicates that exposure of the (111) surface is always favorable, which is similar to the DLA process of snow crystals [27, 28] and plays a critical role for the 2D growth of SiNSs.

Materials with layered structures, such as graphene, Bi_2_Se_3_, and TMDs, grow two dimensionally without the use of catalysts because the *c*-plane surface energy is much lower than that of other surfaces. In contrast with layered crystals, cubic crystals, such as silicon and germanium, are difficult to grow two dimensionally because the surface energy of each plane is not very different and, thus, 2D structures should have higher surface energies than 3D structures due to their higher surface-to-volume ratio. However, our results suggest that such a cubic-structured materials could also achieve 2D growth under DLA environments.

## Conclusions

The 2D growth mechanism of SiNSs was investigated. Single-crystal SiNSs were grown on the substrates using a CVD process under the DLA environments made by a high flow rate of H_2_ and/or Ar gas. Our systematic investigation shows that the DLA environments attribute to the 2D growth of cubic-structured Si by inducing dendritic growth in [110] direction. The environments also attribute to the 2D growth by slow adsorption and rearrangement of the Si atoms on the growth surfaces that makes it possible to achieve thermodynamically stable 2D structure with (111) surface. Our results suggest that 2D growth could be achieved with cubic-structured materials under DLA environments.

## Additional file

Additional file 1:
**Supporting Fiugres. Supporting Figure 1.** Atomic force microscopy images of the SiNSs. **Supporting Figure 2.** SiNSs grown on SiNS.

## Abbreviations

CMOS: complementary metal oxide semiconductor
CVD: chemical vapor deposition
DLA: diffusion-limited aggregation
FET: field-effect transistor
