# Supplementary material for: Growth of Silicon Nanosheets Under Diffusion-Limited Aggregation Environments
Source: Nanoscale Res Lett. 2015 Oct 30;10:429. doi: 10.1186/s11671-015-1138-2 (PMC4627974; doi:10.1186/s11671-015-1138-2)
Supplement: Additional file 1: — Supporting Fiugres. Supporting Figure 1. Atomic force microscopy images of the SiNSs. Supporting Figure 2. SiNSs grown on SiNS. [file 11671_2015_1138_MOESM1_ESM.docx]

**Supporting Figure**


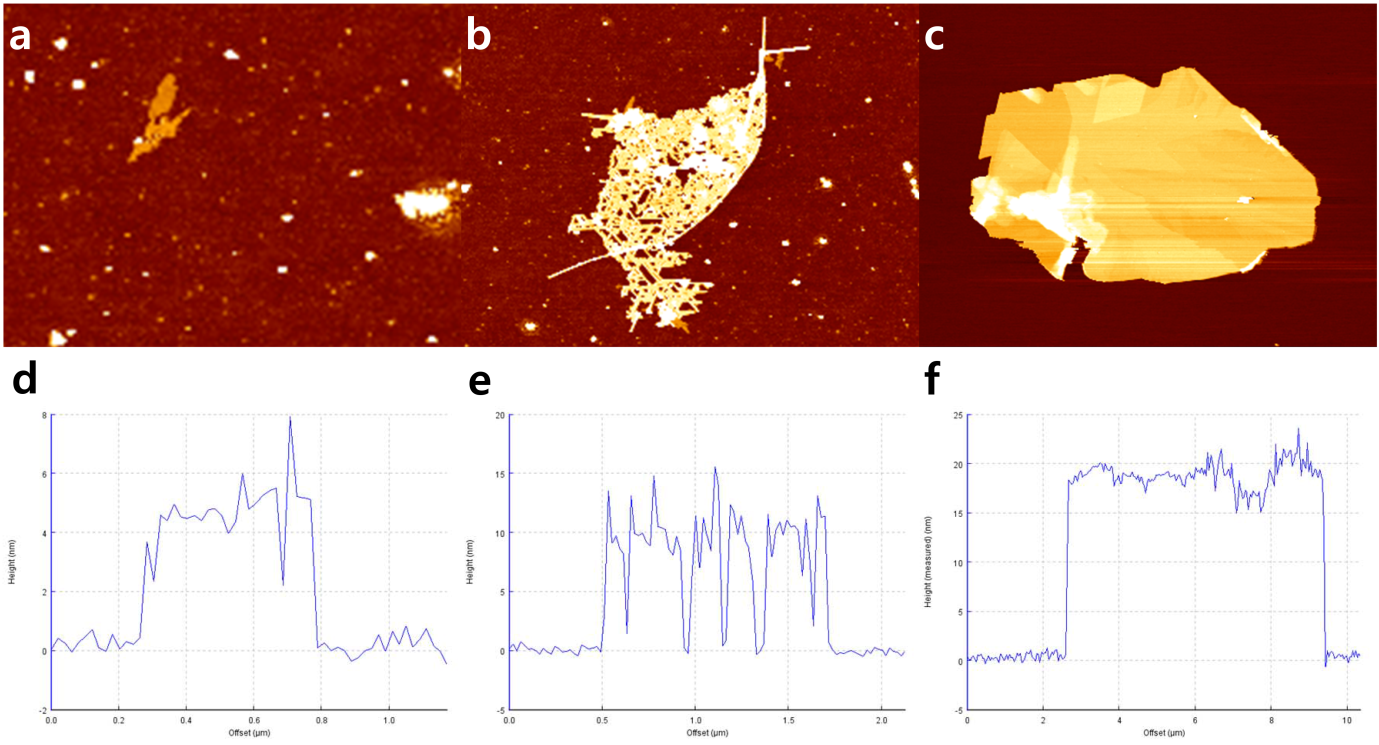


Supporting Figure 1. Atomic force microscopy images of the SiNSs.

**
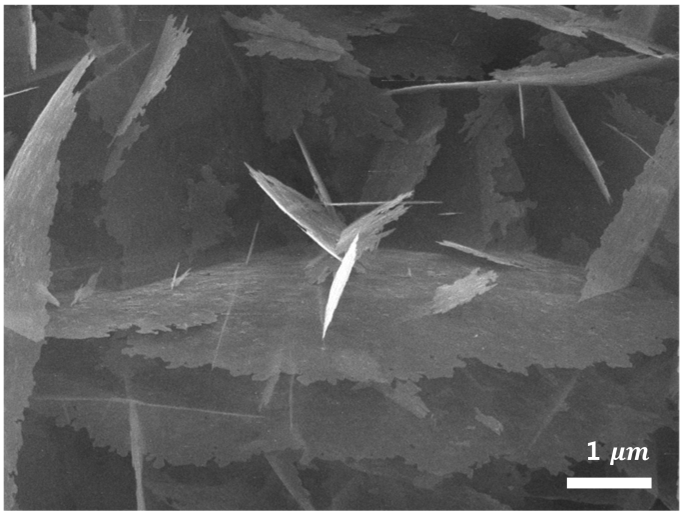
**

Supporting Figure 2. SiNSs grown on SiNS
